# Novel pathophysiological markers are revealed by iTRAQ-based quantitative clinical proteomics approach in vascular dementia

**DOI:** 10.1016/j.jprot.2014.01.011

**Published:** 2014-03-17

**Authors:** Arnab Datta, Jingru Qian, Ruifen Chong, Raj N. Kalaria, Paul Francis, Mitchell K.P. Lai, Christopher P. Chen, Siu Kwan Sze

**Affiliations:** a Department of Pharmacology, Yong Loo Lin School of Medicine, National University of Singapore, Singapore; b School of Biological Sciences, Nanyang Technological University, Singapore; c Institute for Ageing Health, Newcastle University, Campus for Ageing and Vitality, Newcastle upon Tyne, UK; d Wolfson Centre for Age-related Diseases, King's College London, London, UK; e Memory, Aging and Cognition Centre, National University Health System, Singapore

**Keywords:** Vascular dementia, Clinical proteomics, Mass spectrometry, iTRAQ, Vascular dysfunction, Oxidative stress

## Abstract

Vascular dementia (VaD) is a leading cause of dementia in the elderly together with Alzheimer's disease with limited treatment options. Poor understanding of the pathophysiology underlying VaD is hindering the development of new therapies. Hence, to unravel its underlying molecular pathology, an iTRAQ-2D-LC–MS/MS strategy was used for quantitative analysis of pooled lysates from Brodmann area 21 of pathologically confirmed cases of VaD and matched non-neurological controls. A total of 144 differentially expressed proteins out of 2281 confidently identified proteins (false discovery rate = 0.3%) were shortlisted for bioinformatics analysis. Western blot analysis of selected proteins using samples from individual patients (n = 10 per group) showed statistically significant increases in the abundance of SOD1 and NCAM and reduced ATP5A in VaD. This suggested a state of hypometabolism and vascular insufficiency along with an inflammatory condition during VaD. Elevation of SOD1 and increasing trend for iron-storage proteins (FTL, FTH1) may be indicative of an oxidative imbalance that is accompanied by an aberrant iron metabolism. The synaptic proteins did not exhibit a generalized decrease in abundance (e.g. syntaxin) in the VaD subjects. This reported proteome offers a reference data set for future basic or translational studies on VaD.

**Biological significance:**

Our study is the first quantitative clinical proteomic study where iTRAQ-2D-LC–MS/MS strategy has been used to identify the differential proteome in the VaD cortex by comparing VaD and matched control subjects. We generate testable hypothesis about the involvement of various proteins in the vascular and parenchymal events during the evolution of VaD that finally leads to malfunction and demise of brain cells. This study also establishes quantitative proteomics as a complementary approach and viable alternative to existing neurochemical, electron microscopic and neuroimaging techniques that are traditionally being used to understand the molecular pathology of VaD. Our study could inspire fellow researchers to initiate similar retrospective studies targeting various ethnicities, age-groups or sub-types of VaD using brain samples available from brain banks across the world. Meta-analysis of these studies in the future may be able to shortlist candidate proteins or pathways for rationale exploration of therapeutic targets or biomarkers for VaD.

## Highlights

• A quantitative proteomics study to identify the differential proteome in vascular dementia (VaD)
• Identification of 2281 proteins (FDR = 0.3%) from Brodmann area 21 by iTRAQ
• Significant alteration of SOD1, NCAM and ATP5A in VaD compared to matched control
• No generalized decline in the abundance of synaptic proteins (e.g. syntaxin)
• Probable involvement of non-apoptotic modes of cell death in VaD cortex

## Graphical abstract

**Figure:**
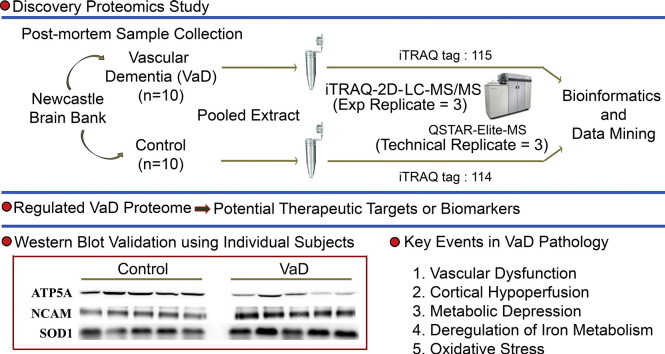

## 1 Introduction

Vascular dementia (VaD) is the second most common cause of age-related dementia after Alzheimer's disease (AD) [1]. Significantly extended life expectancy coupled with the sedentary lifestyle is predicted to increase the incidence of type 2 diabetes, hypertension and hyperlipidemia, all of which are risk factors for vascular disease and subsequent vascular cognitive impairment. The worldwide number of patients with vascular causes of dementia is expected to rise assuming no effective prevention strategies or curative treatments are developed and implemented [2].

Knowledge of VaD pathophysiology has been lagging behind AD for a number of reasons. The lack of suitable animal models and difficulties in obtaining well-characterized clinical samples has hampered the progress of basic and translational research on VaD. Immunohistochemical, neurochemical and electron microscopic studies using post-mortem clinical samples remained the mainstay of mechanistic research [3–5]. Neuroimaging together with clinical observation in patients has provided vital complementary information that forms the basis for the current understanding of VaD [6]. Multiple clinicopathologic substrates have been implicated in the pathogenesis (e.g. cortical or subcortical microinfarcts, demyelination of white matter, cribriform change of basal ganglia and white matter) that links cerebrovascular changes to cognitive impairment, independent of Alzheimer's type pathology [7]. However, the molecular events that drive these vascular and parenchymal changes in the brain are still poorly understood. Given the complexity and heterogeneity of cerebrovascular disorder and the presence of co-morbidities in VaD patients, it is likely that rather than an individual or small number of proteins, multiple candidate proteins present in networks are perturbed leading to the spectrum of cognitive and behavioral symptoms.

The advent of quantitative proteomic technologies using isobaric labeling strategy has made it possible to quantify several proteins in a single experiment for comparative study of global protein regulation across various biological samples. Isobaric tag for relative and absolute quantification (iTRAQ) is one of the most commonly used in vitro isotopic labeling strategies in different areas of biological science and medicine for simultaneously quantifying 4- or 8-plex samples [8]. Clinical proteomics of dementia have flourished in the recent times with a rapid increase in the number of studies analyzing different parts of the post-mortem brain of AD patients for expression profiling studies [9,10]. Apart from the well-known β-amyloid and tau hypothesis, the involvement of pathological events such as excitotoxicity, oxidative stress and inflammation has been highlighted. Post-translational modifications such as carbonylation and phosphorylation of various key proteins have also been found to contribute to AD.

Despite having immense potential to elucidate disease mechanisms, proteomic studies of VaD using preclinical or clinical samples are lacking. Recently, we have successfully applied an iTRAQ-based shotgun neuroproteomic strategy in the area of ischemic stroke to study validated pre-clinical models [11,12], and ischemic infarcts from autopsied human brain [13]. Here, we apply a similar iTRAQ-two dimensional-liquid chromatography–tandem mass spectrometry (iTRAQ-2D-LC–MS/MS) based quantitative proteomic approach on post-mortem VaD and matched non-demented control specimens from Brodmann area 21(BA21) of the temporal lobe for better understanding of the underlying molecular mechanism of VaD. We focused on the temporal lobe because medial temporal lobe atrophy is a common finding in dementia and our recent study suggested that there is a vascular basis for neurodegeneration [14]. The temporal lobe is also relatively free of large infarcts thus making it ideal to detect the survival response of the demented brain [15]. The iTRAQ experiment identified differentially expressed proteins from the pooled lysates of the two groups. Bioinformatics analysis of the proteomic data set revealed the aberrant regulation of proteins related to multiple cellular or subcellular events associated with VaD such as vascular dysfunction and oxidative stress. Some representative deregulated proteins were further validated by Western Blotting (WB) using individual patients. This study is the first quantitative proteomic investigation to reveal the differential global proteomes between VaD and age-matched control brain that provide a valuable data set for future basic or translational studies with individual proteins to propose potential therapeutic targets or biomarkers of VaD.

## 2 Materials and methods

### 2.1 Reagents

Unless indicated, all reagents and assay kits were purchased from Sigma-Aldrich (St. Louis, MO, USA).

### 2.2 Patients and clinical assessments

We obtained frozen brain tissues from 10 non-demented elderly controls and 10 age-matched VaD subjects. The tissues were obtained from the Newcastle Brain Tissue Resource, Institute for Ageing and Health, Newcastle University. Demographic details of the subjects are summarized in Table 1 and can be found for individual subjects in the Supplemental Table 1. For this study, we assessed samples of the grey and white matter from BA21 area of the temporal lobe. VaD was clinically diagnosed using National Institute of Neurological Disorders and Stroke and Association Internationale pour la Recherché et l'Enseignement en Neurosciences criteria [16]. Pathological diagnosis of VaD was defined by the presence of multiple or cystic infarcts involving cortical and sub-cortical structures, border-zone infarcts, lacunae (< 15 mm), micro-infarcts (visible by microscopy only) and small vessel disease in sub-cortical structures in the general absence of neurofibrillary tangles [15]. Clinical evidence of dementia along with the same type or a combination of these lesions at three different coronal levels was considered diagnostic for VaD. None of the VaD cases exhibited tangle burden diagnostic for AD or more than ‘sparse’ neuritic plaques as defined by Consortium to Establish a Registry for Alzheimer's Disease (CERAD) [17,18]. The cognitive assessments were made using Mini-Mental State Examination (MMSE) [19]. MMSE is a 30-point scale to evaluate the cognitive functions that cover orientation, memory, and attention. MMSE also includes the subject's ability to follow verbal and written commands. All control subjects were cognitively normal and they did not have any history of dementia, neurological disease or psychiatric conditions. The control participants did not qualify for the neuropathological diagnostic criteria of AD as none of the subjects had higher than Braak stage III/IV tangle burden or more than “sparse” plaque counts by CERAD criteria. Demographic and disease variables of the control and VaD patients indicated comparable samples for analysis (Table 1; Supplemental Table 1).

Ethical approval was granted by local research ethics committees for this study (Newcastle upon Tyne Hospitals Trust, UK) and permission for post-mortem research using brain tissue was granted for this project. Additional approval for this study was obtained from the Institutional Review Board of the National University of Singapore. Informed consent was also obtained from the guardians of the patients prior to donation of brain tissues.

### 2.3 Experimental design

**Fig. 1:**
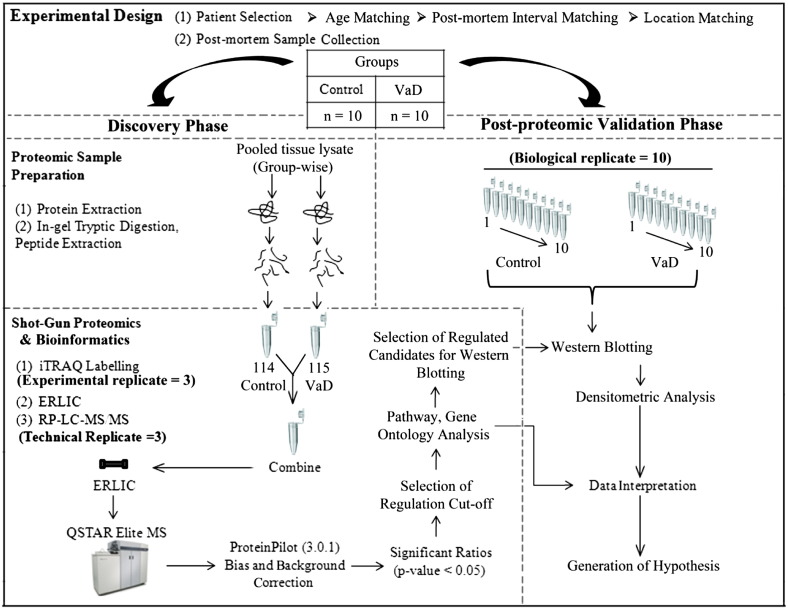
Schematic representation of experimental design showing discovery and validation phase.

The experimental design is described in Fig. 1 and Table 1. Overall, it was divided into two phases, discovery phase and validation phase. The major challenge of expression profiling studies is to discriminate disease-specific changes from individual variations. To discover a consensus proteomic signature predictive of VaD and to gather enough amount of starting material, equal weight of tissues were pooled group-wise from 10 individual samples for the iTRAQ analysis in the discovery phase. Pooling has been suggested to get rid of the outliers or aberrant individual variations while highlighting most consistent disease-specific changes [20]. The tryptic digest from pooled control and VaD (n = 10 for each group) samples were labeled by two iTRAQ tags (114: control, 115: VaD) of the 4-plex iTRAQ kit. The iTRAQ experiment was repeated thrice (experimental replicate = 3, iTRAQ-1, iTRAQ-2, iTRAQ-3) using the pooled digest. Injection for LC–MS/MS was performed thrice (technical replicate = 3) for iTRAQ-1 and iTRAQ-2 and four times (technical replicate = 4) for iTRAQ-3 as multiple injections give better coverage of the target proteome with superior statistical consistency [21]. Thus, a total of 10 (= 3 + 3 + 4) MS injections were performed to generate ten individual data sets that will allow for the calculation of technical as well as experimental variations arising from individual iTRAQ experiments. Post-proteomic validation of selected proteins from the iTRAQ data set was performed on individual patients (biological replicate = 10 for control and VaD group) using a separate tissue specimen from the BA21 area of the same individual by WB analysis to measure the extent of biological variation.

### 2.4 Proteomics

#### 2.4.1 Sample preparation

Sample processing including centrifugation and sonication were performed at 4 °C unless mentioned otherwise. A biphasic chloroform/methanol extraction coupled with acetone precipitation was adopted with slight modifications as described previously [22,23]. This is to maximize the recovery of various macromolecules and matabolites from scarce clinical specimens suitable for downstream processing through different -omics platforms. Briefly, equal quantities of frozen tissues (w/w) were pooled in a group-wise manner and grounded into fine powder using liquid nitrogen before being suspended in a mixture of methanol (Merck KGaA, Darmstadt, Germany), chloroform and water (J.T. Baker, Center Valley, PA, USA) (1:1:2). The suspension was mixed for 1 h and subsequently centrifuged (15 000 ×*g*, 2 min) to obtain the pellet. The non-polar chloroform fractions were kept for separate studies. The previous step was repeated using a mixture of methanol, chloroform and water (4:1:3) for washing the pellet. The polar methanol–water phase was combined from consecutive steps and mixed with acetone (overnight, 4 volumes of acetone, − 20 °C) followed by centrifugation (15 000 ×*g*, 5 min) to precipitate the residual proteins. Methanol was used subsequently to wash the protein containing pellets and remove contaminants that were followed by centrifugation (15 000 ×*g*, 2 min). Protein pellets were dissolved in 8 M urea with protease inhibitor cocktail (Complete (Roche; Mannheim, Germany)) by intermittent sonication for 20 s (amplitude, 30%) using a Vibra Cell high intensity ultrasonic processor (Jencon Scientific Ltd.; Leighton Buzzard, Bedfordshire, U.K.). It was subsequently centrifuged (20 000 ×*g* for 30 min) to collect the supernatant. The protein concentration of the supernatant was determined by the bicinchoninic acid assay.

#### 2.4.2 In-gel tryptic digestion and isobaric labeling

The samples (250 μg of proteins/condition) were then reduced (5 mM tris-(2-carboxyethyl) phosphine, 30 °C, 3 h) and alkylated (20 mM methyl methanethiosulfonate in isopropanol, room temperature, 45 min in dark) followed by a two-step digestion (16 h + 4 h) at 30 °C with sequencing-grade modified trypsin (Promega, Madison, WI, USA) at a ratio of 1:100 (trypsin: sample) in 50 mM triethylammonium bicarbonate (TEAB). The tryptic digested peptides were desalted using Sep-Pak C18 cartridges (Waters, Milford, MA, USA) and dried in a vacuum centrifuge. The dried peptides were reconstituted into 0.5 M TEAB and labeled with respective isobaric tags of 4-plex iTRAQ reagents (Applied Biosystems, Foster City, CA, USA) as follows: control, 114; VaD, 115 (Fig. 1). The labeled peptides were combined and dried in a vacuum centrifuge. The iTRAQ sample was desalted using Sep-Pak C18 cartridges and dried before fractionation. Three batches of experimental replicates were prepared separately as showed in the experimental design.

#### 2.4.3 Electrostatic repulsion and hydrophilic interaction chromatography (ERLIC)

The mixture of iTRAQ-labeled peptides was fractionated using a PolyWAX LP anion-exchange column (4.6 × 200 mm, 5 μm, 300 Å) (PolyLC, Columbia, MD, USA) on a Prominence UFLC system (Shimadzu, Kyoto, Japan) and monitored at the wavelength of 280 nm. Thirty five fractions were collected during a 60 min gradient of 100% buffer A (0.1% acetic acid, and 10 mM ammonium acetate in 85% acetonitrile (ACN)) for 5 min, 0–36% of buffer B (0.1% formic acid (FA) in 30% ACN) for 25 min, 36–100% of buffer B for 20 min, and 100% of buffer B for the last 10 min at a flow rate of 1 ml/min [24,25]. Eluted fractions were pooled into 26 fractions depending on the peak intensities. They were dried in a vacuum centrifuge and redissolved in 0.1% FA in 3% ACN for LC–MS/MS analysis.

#### 2.4.4 Reverse phase LC–MS/MS analysis using QSTAR

Each fraction of redissolved iTRAQ-labeled peptides was sequentially injected in triplicate and separated in a home-packed nanobore C18 column with a picofrit nanospray tip (75 μm ID × 15 cm, 5 μm particles) (New Objectives, Woburn, MA, USA) on a Tempo nano-MDLC system coupled with a QSTAR Elite Hybrid MS (Applied Biosystems/MDS-SCIEX). Each fraction was independently analyzed by the LC–MS/MS over a gradient of 90 min with the constant flow rate of 300 nl/min. Data acquisition in QSTAR Elite was set to positive ion mode using Analyst QS 2.0 software (Applied Biosystems). The precursors with a mass range of 300–1600 m/z and calculated charge from + 2 to + 5 were selected for fragmentation. For each MS spectrum, 5 most abundant peptides at most above a 5-count threshold were selected for MS/MS, and the selected precursor was dynamically excluded for 20s with a mass tolerance of 0.1 Da. Smart information-dependent acquisition was activated with automatic collision energy and automatic MS/MS accumulation. The fragment intensity multiplier was set to 20 and maximum accumulation time was 2 s.

#### 2.4.5 Mass spectrometric raw data analysis

Spectra acquired from each of the technical and experimental replicates were submitted alone and together to ProteinPilot Software (v 3.0, Revision Number: 114 732, Applied Biosystems) for peak list generation, protein identification and quantification against the concatenated target-decoy Uniport human database (downloaded on 12 March 2012). User defined parameters in ProteinPilot software were configured as described previously [11] with minor modifications as follows: (i) Special factors, urea denaturation; (ii) Specify Processing, quantitate, bias and background correction. Default precursor and MS/MS tolerance for QSTAR ESI-MS instrument were adopted automatically by the software. The false discovery rates (FDR) of both peptide and protein identification were set to be less than 1% (FDR = 2.0 ∗ decoy_hits / total_hits).

#### 2.4.6 Bioinformatics analysis

The open-source software Panther and David were used for the enrichment analysis in various categories like Gene Ontology and pathways by submitting Uniprot accession numbers of the short-listed proteins [26,27]. The human proteome was used as a default background for David. The enrichment analysis in David identifies overrepresentation of certain phenotype (i.e. a group of proteins common to a specific pathway, class, location, function, disease or any other attribute) from long list of regulated proteins that did not occur only by chance. Significances were expressed by fold of enrichment and *p*-value before and after corrections with Benjamini–Hochberg or modified Fisher's exact test. Any phenotype was considered enriched when the *p*-value was less than 0.01 and FDR less than 1% after corrections with both the above-mentioned methods.

#### 2.4.7 Post-proteomic validation

##### 2.4.7.1 Sample processing

The samples were briefly thawed on ice and dissected to remove meninges and exclude the blood vessels and the white matter. Cleaned samples were then homogenized at 50 mg tissue/ml in an ice cold cell lysis buffer (20 mM Tris–HCl, 150 mM NaCl, pH 7.5) (Cell Signaling Technology, Danvers, MA, USA). Protease inhibitor cocktail (Complete (Roche)) was added immediately before use. Protein concentrations were measured using a 2-D Quant kit (Amersham Biosciences, Piscataway, NJ, USA).

##### 2.4.7.2 WB analysis

WB was performed after SDS-PAGE by probing with primary antibodies at the indicated dilutions: anti-ACTB (beta-actin, 1:10 000, mouse monoclonal; Millipore, Billerica, MA, USA), anti-BCL2 (B-cell CLL/lymphoma 2, 1:1000, mouse monoclonal; Santa Cruz Biotech, Santa Cruz, CA, USA), anti-ferritin (1:4000, rabbit polyclonal; Abcam, Cambridge, UK), anti-HSPA4 (heat shock 70 kDa protein 4, 1:5000, mouse monoclonal; Abcam), anti-ICAM5 (intercellular adhesion molecule 5, 1:1000, rabbit polyclonal; Abcam), anti-NCAM (neural cell adhesion molecule, 1:10 000, rabbit polyclonal; Santa Cruz Biotech), anti-PEA15 (phosphoprotein enriched in astrocytes, 1:800, rabbit polyclonal; Abcam), anti-SOD1 (superoxide dismutase 1, 1:2000, rabbit polyclonal; Abcam), anti-syntaxin (1:10 000, mouse monoclonal; Milipore), anti-SYNPO (synaptopodin, 1:1000, rabbit polyclonal; Abcam) and anti-VDAC1 (voltage-dependent anion channel 1, 1:5000, mouse monoclonal; Santa Cruz Biotech). Immunoblotting of ACTB was used as a loading control. Apart from that, two commercially available antibody cocktails were used; Mitoprofile Total OXPHOS Human WB Antibody Cocktail (1:2000, mouse monoclonal, MS601; Mitoscience, Eugene, Oregon) and Apoptosis Western Blot Cocktail (1:250, MS1107; Mitoscience). MS601 consisted of five antibodies targeting key proteins of oxidative phosphorylation (i.e. NADH dehydrogenase [ubiquinone] 1 beta subcomplex subunit 8, mitochondrial (NDUFB8); Cytochrome c oxidase subunit 2 (MT-CO2), Succinate dehydrogenase [ubiquinone] iron–sulfur subunit, mitochondrial (SDHB); Cytochrome b-c1 complex subunit 2, mitochondrial (UQCRC2) and ATP synthase subunit alpha, mitochondrial (ATP5A)). MS1107 detects 32 kDa pro-caspase 3, 17 kDa active caspase 3, 89 kDa apoptosis-specific cleaved Poly [ADP-ribose] polymerase 1 (PARP) along with muscle actin which is incorporated as a loading control. This cocktail does not react with full-length PARP. Twenty to forty micrograms of proteins were used for WB depending on the sensitivity of the specific antibody. Immunoreactivity was detected by using an HRP chemiluminescent substrate reagent kit (Invitrogen, Carlsbad, CA). A pooled sample was used to normalize the inter-gel variation between repeated runs for the same protein.

##### 2.4.7.3 Statistical analysis

All statistical analyses were performed using SPSS 13.0 for Windows software (SPSS Inc.). Normality of data was first checked using the Kolmogorov–Smirnov test. Independent sample *t*-test was used to compare the means of control and VaD group. Equal variances were assumed for the calculation of *p*-values as the two groups had equal number of subjects (n = 10). Non-parametric Mann–Whitney *U* Test was used for comparing non-normal distributions. Correlations involving only continuous data (i.e. age, post-mortem interval, and protein abundance) were analyzed by Pearson's product moment correlation. Spearman's rank correlation was used when one of the variables are ordinal in nature (i.e. MMSE score, Braak staging, and CERAD score). Experimental data for WB analysis were presented as mean ± SEM. Statistical significance was accepted at *p < 0.05 and **p < 0.01.

## 3 Results

To understand the global proteomic change in VaD, pooled tissue extracts were compared between groups of age-matched control and VaD subjects by iTRAQ experiment.

### 3.1 Quality control of iTRAQ data set

A strict cut-off of unused ProtScore ≥ 2 was used as the qualification criteria to minimize the false positive identification of proteins. With this criteria, 1569, 2039 and 1525 proteins were identified from the three iTRAQ experiments with a FDR of < 0.8%. To improve the confidence of identification and quantification, all ten individual injections from the three iTRAQ experiments were combined for subsequent analysis. The combined data set had 2281 proteins with a FDR of 0.3% using the ProtScore cut-off mentioned above (Supplemental Table 2). The average number of unique peptides (having a confidence level of ≥ 95%) detected per protein was 21.8. More than 55% of the proteins (1260 proteins) had ≥ 5 unique peptides, while around 14% of proteins were identified with a single peptide. The average % coverage for the combined data set was 28.2%, whereas around 39.4% of the proteins (901 proteins) had % coverage more than the average level. Intriguingly, some proteins were identified with 100% sequence coverage (e.g. Hemoglobin subunit beta (HBB), Gamma-synuclein, and Fructose-bisphosphate aldolase C) (Supplemental Table 2). This result is better than our previously published report where a single iTRAQ experiment was performed to study the regulated proteome [12]. To the best of our knowledge, this data set is likely the biggest neuroproteomics data set from similar iTRAQ based quantitative clinical proteomic study. Next, the ratios were sorted using a *p*-value cut-off of 0.05 to shortlist a set of 171 significantly perturbed proteins for further analysis.

### 3.2 Estimation of threshold for confidently defining perturbed proteins

**Fig. 2:**
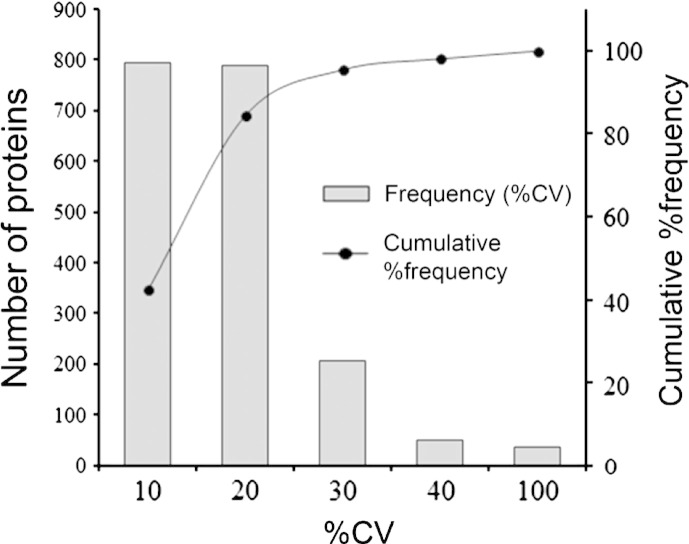
Percent variation in iTRAQ ratios (115/114) between the same protein found in various technical and experimental replicates following MS. The primary vertical axis represents the corresponding number of the proteins (bars) having different % co-efficient of variation (%CV) that was plotted in the horizontal axis. The secondary vertical axis represents the cumulative % of the counted proteins (lines) where 100% equals to 1878 proteins. These proteins have confidently been identified in at least 3 out of 10 MS runs. Ninety eight percent of the proteins had less than 40% of %CV. Accordingly; the regulation cut-off was set at 1.4-fold.

To determine the cut-off for up- or down-regulation, the deviation of ratios (i.e. 115/114) of individual proteins between various technical and experimental replicates were measured as described previously [11,12]. The proteins that have been confidently identified in at least 3 injections were included. Accordingly, geometric mean, standard deviation and %coefficient of variation (%CV) of 1878 proteins were determined. The average %CV was around 13%. The %CV was less than 20% and 40% for more than 84% and 98% of the proteins (Fig. 2). Based on this, the regulation threshold was set at 1.4 fold; ratio > 1.40 or < 0.71 was considered as up- or down-regulated respectively. Applying this threshold to the 171 significantly perturbed proteins, a list of 144 proteins (6.3% of initial hits) was obtained; where 72 each were up- and down-regulated (Supplemental Table 3). This was advanced to the next stage for bioinformatics analysis to retrieve hidden biological trends.

### 3.3 Bioinformatics analysis

Bioinformatics analysis revealed up-regulation of various neurotransmitter signaling pathways (e.g. glutamate and acetylcholine) while glycolysis, tricarboxylic acid cycle, ATP synthesis and pyruvate metabolism were featured among the down-regulated pathways. Recently, the up-regulation of GluR2 subunit of glutamatergic α-amino-3-hydroxy-5-methyl-4-isoxazolepropionic acid receptor was reported in the BA22 area of the VaD brain when compared with age-matched control and mixed AD/VaD cases, which is concordant with the trends observed in our study [28]. Proteins related to metabolic, immune system process, cell adhesion and apoptosis exhibited mixed trends. Majority of metabolic proteins (77.9%, 60/77) were decreased in the VaD brain, while proteins linked to cell adhesion (90.9%, 10/11) and immune system processes (61.1%, 11/18) were elevated. This is consistent with a recent RNA microarray study reporting the up-regulation of genes associated with cell adhesion and immune response in the deep subcortical white matter lesion [29]. The levels of most of the perturbed extracellular proteins (87.5%, 7/8) including FGB, COL6A3, NCDN, VCAN, and NFASC were increased. Among the perturbed protein classes, the calcium binding proteins (80%, 4/5; SLC25A3, SPTBN2, SPTBN1, PLEC) and chaperones (71.4%, 5/7; HSPB1, HSPA5, STIP1, CCT8, TBCA) were mainly elevated while all proteases (5/5; e.g. NPEPPS, PREP and PSMA6) were reduced in the VaD brain. Finally, guided by the above trends, the selected list of proteins was manually classified into various functional categories with additional proteins taken from the complete data set based on their relevance with the respective categories (Table 2).

### 3.4 Post-proteomic validation by WB analysis

**Fig. 3:**
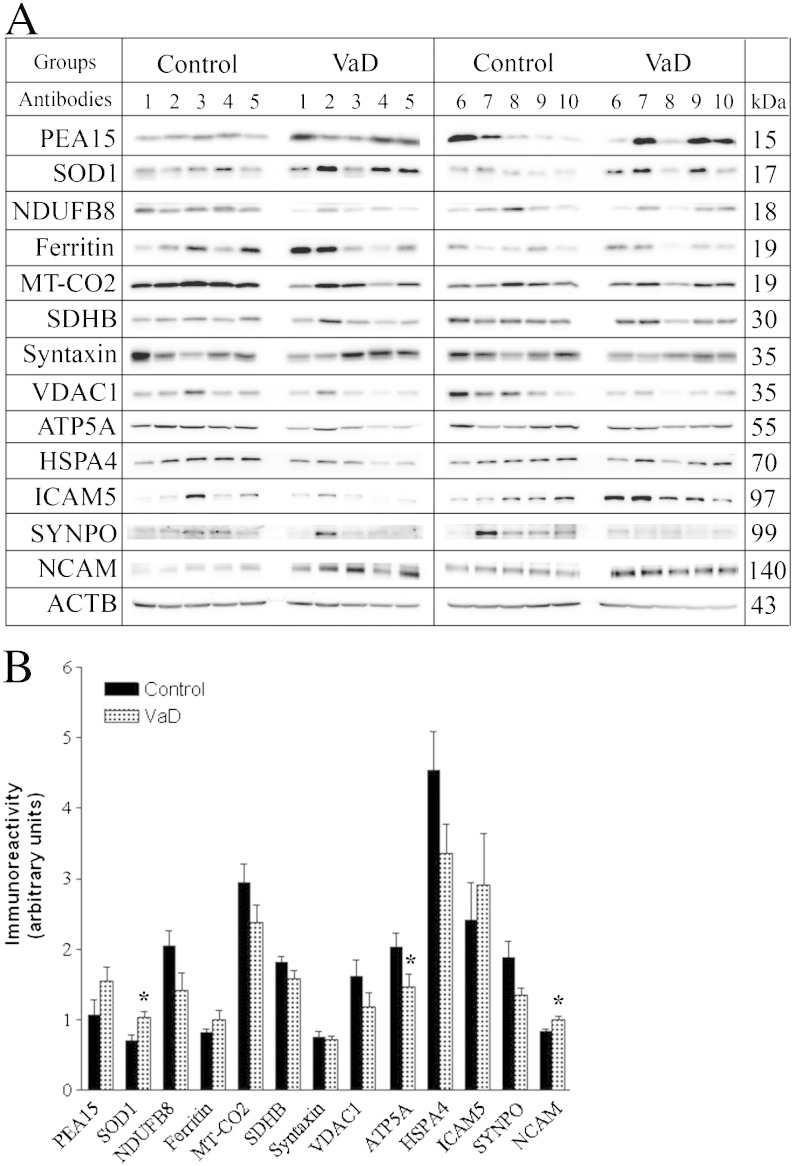
Post-proteomic validation of the selected proteins using individual patients from control and VaD groups by WB analysis. Equal amount of protein was loaded as measured by the 2D Quant kit. A) Representative immunoblots showing the protein levels in B21 area of all twenty patients (n = 10 per group). Details of the patients can be found in the Supplemental Table 1. ACTB was used as a loading control. B) Bar chart of densitometric analysis for comparing the protein expression levels by the statistical analysis. SOD1 and NCAM were significantly increased whereas ATP5A was reduced significantly in the VaD brain. Trends were observed for SYNPO, HSPA4, VADC1, ferritin, PEA15 and ICAM5 without reaching a statistical significance. Data was presented as mean ± SEM (n = 10), where *p < 0.05, significantly different from control using independent-sample *t*-test.

Eleven proteins (i.e. SOD1, NCAM, ATP5A, UQCRC2, SYNPO, HSPA4, VDAC1, ferritin, PEA15, ICAM5 and syntaxin) were selected from the list of 171 significantly regulated proteins for validation in twenty subjects individually (Fig. 3). They were selected from a wide range of molecular functions according to the bioinformatics analysis, such as energy metabolism (e.g. ATP5A, UQCRC2), oxidative stress (e.g. SOD1, ferritin),inflammation (e.g. NCAM, ICAM5), synaptic transmission (e.g. SYNPO, syntaxin) and apoptosis (e.g. HSPA4, PEA15, VDAC1). In addition, three more candidates (i.e. SDHB, MT-CO2 and NDUFB8) related to oxidative phosphorylation were also included as negative control to check the reliability of the relative quantization as none of them were significantly (*p* > 0.05) regulated in the proteomics result (Table 2). It should be noted here that unlike iTRAQ experiment, which was performed on pooled lysates, the *p*-value in the WB analysis is an estimate of the biological variation based on the cohort of twenty individuals.

The abundance of SOD1 (*p* = 0.014) and NCAM (*p* = 0.018) was significantly higher whereas ATP5A (*p* = 0.047) and UQCRC2 (*p* = 0.006) were significantly lower in VaD compared to the controls. SYNPO (*p* = 0.062), HSPA4 (*p* = 0.100) and VDAC1 (*p* = 0.168) showed a decreasing trend unlike ferritin (*p* = 0.192), PEA15 (*p* = 0.114) and ICAM5 (*p* = 0.578) that showed an increasing trend in the VaD group. A poor *p*-value for WB analysis is related to higher individual variation in the expression of a protein within the same group. Hence, increasing the number of subjects per group could have resulted in a statistically significant difference for some of the above mentioned candidates. Considering the groups were not gender-matched, individual abundances of all proteins from WB results were compared between male (n = 9) and female (n = 11) to evaluate the influence of gender as a potential confounding factor. The levels of UQCRC2 were found to be significantly lower (*p* = 0.027) in male compared to female subjects thus excluding it from further discussion.

Syntaxin did not exhibit any difference between VaD and control groups by WB, although it was found to be up-regulated (i.e. STX1A and STX1B, ~ 1.8 fold) in the iTRAQ analysis (Table 2). This ambiguity can be related to the sampling of an independent piece of tissue for proteomic and WB validation experiment or may have arisen from the different sample processing protocols. The three negative controls (SDHB, MT-CO2 and NDUFB8) did not show difference between VaD and control brain tissues in both WB and iTRAQ, indicating the reliability of the sample pooling strategy in the discovery phase. Overall, a consistent trend was observed with the iTRAQ result for the selected proteins with the exception of syntaxin.

### 3.5 Correlation analyses

One of the major confounding factors for studies involving post-mortem specimens of geriatric subjects is sampling artifact that may alter pattern or magnitude of protein expression [30]. Correlation analysis was performed for two groups of subjects individually and in combination in order to study linear associations between the immunoreactivities of the key perturbed proteins and available neuropathological or clinical variables. Individual abundances of none of the proteins used for WB validation correlated with age or post-mortem interval of the recruited subjects thereby excluding the presence of systematic bias related to the sample collection. Moderate to strong correlations were obtained in between the abundances of various proteins selected for validation. Notably, immunoreactivities of two significantly elevated proteins in the VaD brain (i.e. SOD1 and NCAM) were positively correlated with various mitochondrial proteins such as VDAC1 (SOD1, r = 0.86, *p* < 0.01), SDHB (SOD1, r = 0.76, *p* < 0.05; NCAM, r = 0.65, *p* < 0.05), and MT-CO2 (SOD1, r = 0.71, *p* < 0.05; NCAM, r = 0.80, *p* < 0.01) and were negatively correlated with the synaptic protein, syntaxin (SOD1, r = − 0.77, *p* < 0.05; NCAM, r = − 0.69, *p* < 0.05). No association was found between the protein levels and MMSE scores for the VaD group. Detailed results of the correlation analysis can be found in the Supplemental Table 4.

## 4 Discussion

To our knowledge, this is the first comparative proteomic profiling of post-mortem brain tissues of VaD and control subjects by combining the attributes of biphasic chloroform/methanol extraction for sample preparation, multiplex isobaric labeling with iTRAQ reagent chemistry, two dimensional liquid chromatography, nano-electrospray ionization and high resolution tandem mass spectrometry (iTRAQ-2D-LC–MS/MS).

### 4.1 Unraveling the pathophysiology of VaD — a systems biological perspective

To ensure specificity of dementia due to vascular causes and not due to the presence of co-morbidities, extensive neuropathological characterization was performed to exclude confounding from two other possible causes of dementia (e.g. AD and dementia with Lewy bodies (DLB)). VaD samples did not exhibit significant Alzheimer's type of pathology as the neuritic plaques were confined to only the entorhinal areas (stage I–II) (Table 1). These stages are generally considered to be without symptoms or be associated with little evidence of dementia [31]. Additionally, immunoreactivity of none of the tested fourteen candidates' correlated with the Braak staging (n = 17), MMSE score (n = 7) and CERAD score (n = 10) thus excluding probable etiological association between key perturbed proteins with Alzheimer's pathology (Supplemental Table 4). Neuropathological examination also confirmed the absence of Lewy body pathology in the selected subjects (data not shown). Hence, the presence of cognitive impairment as seen by the low MMSE scores in the VaD group (Table 1) was associated with cerebrovascular pathology through the deregulation of different functional classes of proteins found in the current data set.

### 4.2 Cortical hypoperfusion and vascular dysfunction in VaD

**Fig. 4:**
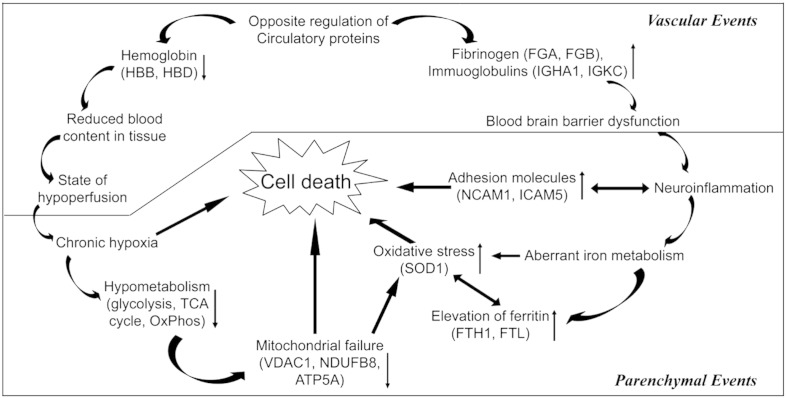
Schematic diagram showing the interplay of various vascular and parenchymal events during the evolution of VaD. ↑, up-regulation; ↓, down-regulation.

The plasma proteins in our short-listed data set may allow better understanding of the perfusion status of the brain in VaD subjects. The significantly up-regulated list of proteins in the proteomic data set was dominated by different isoforms of immunoglobins (e.g. IGHA1, IGHG2, IGSF8, IGLC2 and IGKC) and other known plasma proteins (e.g. FGB). Intriguingly, HBB was the protein with maximum fold of down-regulation. HBD also showed significant down-regulation albeit with lesser magnitudes (Table 2, Fig. 4). Hemoglobins (i.e. HBB and HBD) generally remained entrapped in the erythrocytes, which does not extravasate beyond the circulation unless there is a hemorrhage. The decreased presence of hemoglobin may be indicative of a perfusion failure in the VaD cortex along with a diffuse leakage of the blood brain barrier (BBB) that is manifested by an apparent increase in the levels of several proteins of circulatory source. In addition, we observed significant down-regulation of a key component of oxidative phosphorylation (i.e. ATP5A) while HSPA4 and mitochondrial protein VDAC1 showed down-ward trends during WB validation indicating a state of hypometabolism and mitochondrial failure (Fig. 3). Further, the up-regulation of an isoform of collagen (i.e. COL6A3) in the iTRAQ result may indicate accumulation of extracellular matrix components that could cause collagenosis and vascular stenosis thus aggravating the perfusion failure [32]. The unraveled molecular events from our unbiased quantitative proteomic study may offer a mechanistic explanation for previous observations from neuroimaging and neuropathologic studies. The disruption of BBB in small vessel disease has been documented using dynamic contrast-enhanced magnetic resonance imaging in live patients and through immunohistochemistry of immunoglobulins and fibrinogen of brain tissue [33,34]. Cortical hypometabolism and hypoperfusion have been detected with functional imaging like positron emission tomography in patients of VaD in comparison with healthy controls [35] and by two-dimensional xenon-133 inhalation technique in the ipsilateral fronto-parietal cortex in the presence of sub-cortical infarcts [36].

Considering chronic leakage of fluid and macromolecules, a likely consequence will be induction of an inflammatory response in the temporal cortex of VaD brain. Subsequently, curating the proteomic data set for other hallmarks of inflammation identified few adhesion molecules (e.g. L1CAM, NCAM1, ICAM5) showing an up-regulation in the VaD group (Table 2, Figs. 3, 4). The decrease in HSPA4, which normally functions as a chaperone and protects cells from inflammation during cerebral ischemic injury, may further worsen the inflammatory condition [37]. The levels of L1CAM and NCAM were found to be higher in the cerebrospinal fluid (CSF) of VaD, AD and MIX dementia patients compared to non-demented controls [38]. Soluble form of ICAM5 has been detected in the serum within the first 48 h of cerebral hypoxic–ischemic injury of mice, where shredded neuronal ICAM5 could act as an anti-inflammatory agent by suppressing the T-cell activation [39,40].

### 4.3 Deregulation of iron metabolism and oxidative stress

In the human brain, iron is ubiquitous and is responsible for the activity of key enzymes (e.g. cytochromes, mitochondrial non-haem iron proteins) and for the synthesis of neurotransmitters. Most of the total iron in a healthy brain is sequestered by ferritin thus limiting its pro-oxidant potential. Brain ferritin-associated iron levels increase with age. Abnormal elevation of brain iron and ferritin has been reported in the neurodegenerative dementias (i.e. AD, PD and dementia with Lewy Bodies) suggesting that increased iron levels may contribute to the misfolding and subsequent deposition of key proteins [41]. Although consistent correlation between iron accumulation and cognitive dysfunction has been documented in animal or clinical studies related to neurodegenerative diseases like AD, similar reports are scarce in the area of VaD [42]. Here, we report an increase of brain ferritin (H- and L-isoforms) levels of VaD patients compared to the age-matched control group (Table 2, Fig. 3). Interestingly, out of the tested fourteen candidates for validation, only the levels of ferritin (r = 0.76, *p* < 0.05) positively correlated with the duration of illness in the VaD group (n = 8) indicating a probable increase in iron accumulation along with the progression of VaD (Supplemental Table 4).

**Fig. 5:**
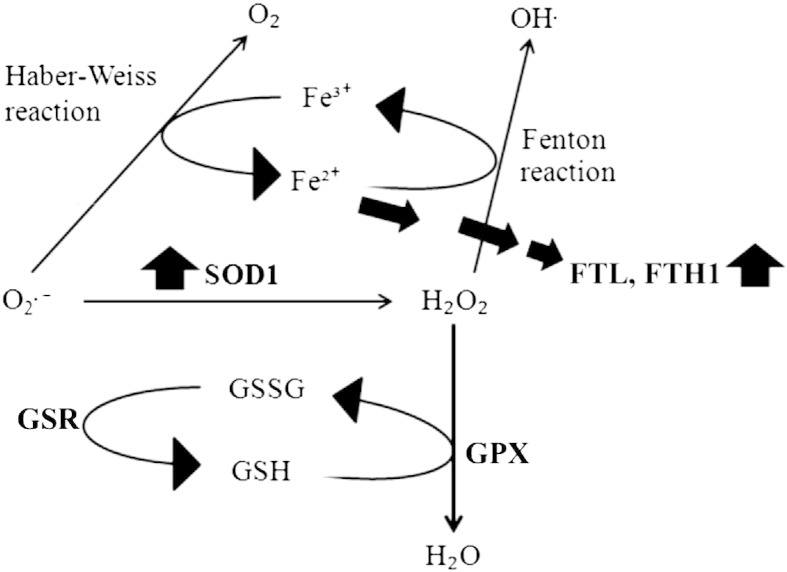
Schematic diagram showing the probable involvement of SOD1 and ferritin (FTL, FTH1) in detoxifying the demented brain cells from iron overload and oxidative imbalance.

Considering that increased level of ferritin may help the demented brain to adapt against the oxidative stress by capturing the free iron, the significant up-regulation of SOD1 in VaD brain could have a complementary protective role. SOD1 catalyzes the dismutation of superoxide anion (O_2_^−^) into oxygen and hydrogen peroxide (H_2_O_2_). The O_2_^−^ can react with ferric iron (Fe^3 +^) by Haber–Weiss reaction to produce ferrous iron (Fe^2 +^) which further reacts with H_2_O_2_ by Fenton reaction to generate highly reactive and damaging hydroxyl radical (OH^−^) (Fig. 5). Hence, both ferritin and SOD1 could work in tandem to remove ferrous iron (Fe^2 +^) and O_2_^−^ radicals thereby preventing oxidative injury in the demented brain. Nevertheless, we also observed an upward trend for GPX in the proteomic data set, an enzyme that reduces H_2_O_2_ to water using reduced glutathione (GSH). Several studies with animal models of ischemic stroke (focal or global cerebral ischemia) have demonstrated the neuroprotective potential of SOD1 [43]. In line with that, we have seen reduced levels of SOD1 and elevated levels of ferritin in the ischemic infarcts (i.e. core of injury), when compared with matched control subjects [13]. Recently, SOD1 has been selected to genetically modify neural stem cells as its overexpression has improved their survival following intracerebral administration in rodent models of ischemic stroke. This is consistent with the probable protective role of SOD1 as discussed above [44].

### 4.4 Mechanisms of cell death in VaD

**Fig. 6:**
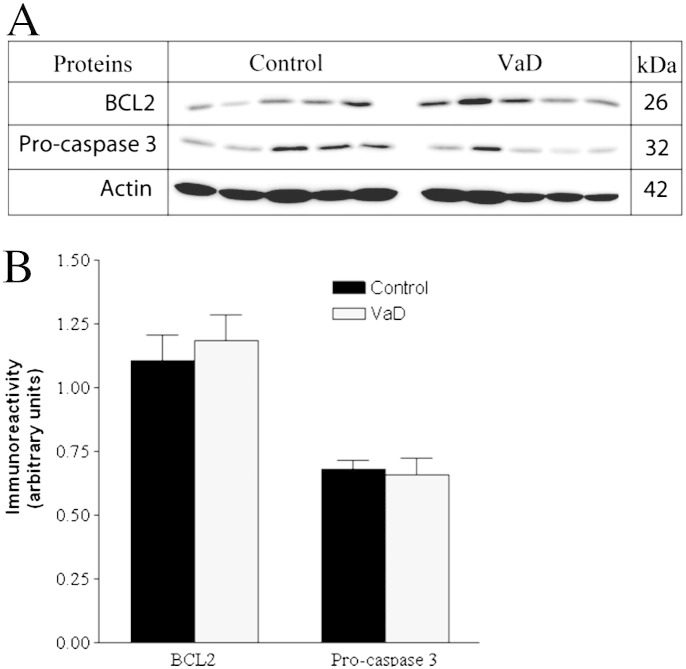
A. Representative immunoblots showing the levels of important markers of apoptosis in the temporal cortex of control and VaD patients. B. Bar chart of band densities (in arbitrary units) involving all twenty individuals and normalized with the expression of muscle actin, which was used as a loading control. There were no statistically significant difference in the abundance of BCL2 and pro-caspase 3 between control and VaD group. Data was presented as mean ± SEM (n = 10), where *p < 0.05, significantly different from control using independent-sample *t*-test.

Inflammatory and oxidative stress mechanisms as discussed above are suggested to precede apoptosis that finally causes cell death in the demented brain [7,29]. Recent studies have demonstrated neuronal apoptosis as one of the contributing pathways of cell death in the neocortex of cerebral autosomal-dominant arteriopathy with subcortical infarcts and leukoencephalopathy [45]. Hence, to elucidate the pathways that seal the fate of the stressed cells in this group of VaD patients, we had tested the expression of well-known apoptotic markers that include pro-caspase 3, p17 subunit of active caspase 3, cleaved PARP along with anti-apoptotic BCL2 protein. Pro-caspase 3 forms activated caspase 3 at the initiation of apoptotic cascade that proteolytically cleaves the DNA repair enzyme, PARP to generate the 89 kDa apoptosis-specific PARP fragment. However, no significant differential expression of pro-caspase-3 and BCL2 between VaD and control subjects was observed (Fig. 6). The PARP fragment and p17 subunit of caspase 3 remained undetected in either group following WB analysis indicating the absence of significant degradation of the PARP enzyme by active caspase 3. Therefore, our data points toward the involvement of non-apoptotic pathways of cell death in the cortex of the studied VaD patients. However, there is tantalizingly little data on the underlying mechanisms of cell death in VaD and the participation of autophagy has been speculated [7].

Overall, our quantitative proteomic approach documents the up-regulation of seemingly protective and deleterious pathways simultaneously in the temporal cortex of the VaD subjects. An inflammatory condition (i.e. NCAM1) or vascular insufficiency (e.g. HBB) and a decline in the energy metabolism (i.e. ATP5A1) are accompanied by the up-ward trend for anti-inflammatory (i.e. ICAM5), anti-apoptotic protein (i.e. PEA15) or proteins related to iron storage (i.e. FTL, FTH1) or anti-oxidative function (i.e. SOD1) (Fig. 4). This penumbra-like condition is not surprising considering the presence of predominant sub-cortical lesions in these VaD subjects as we deliberately sampled one neocortical area (i.e. BA21) that is located at a distance from the epicenter of the vascular injury. Accordingly, the synaptic proteins (e.g. syntaxin) did not show a unanimous reduced expression indicating no generalized synaptic decline as seen in the case of AD affected brain [5]. Hence, our proteomic result was able to identify the key molecular substrates behind different pathological processes that work in concert to cause the demise of the brain cells in VaD. Further studies on larger cohort of patients and with pre-clinical models could better explain these aberrant regulations and their interactions with the existing supportive therapies of VaD like cholinesterase inhibitors, antioxidants or vasodilators.

## 5 Limitations

Being a study performed using PM specimens; the data interpretation is limited by the availability of samples. The groups were not matched by sex and gender was found to be a confounding factor for one of the candidate proteins (i.e. UQCRC2) shortlisted for WB validation. However, all other proteins including pro-caspase-3 and BCL2 did not show significant difference between male and female groups. The age of onset of VaD is variable among individuals along with their different lifestyle and genetic backgrounds. Details of co-morbidities, risk factors, medications and exact cause of death are not available. Despite these limitations, they had similar clinical and neuropathological presentations in a group-wise manner that made the proteomic comparison of pooled samples of identical brain location possible.

Our experimental design did not include groups of subjects with clinically or biochemically overlapping pathologies such as AD, DLB or ischemic stroke for a comparative validation of the short-listed candidates. Hence, our conclusions are necessarily limited to VaD as we are unable to comment on the specificity of the reported candidates in the context of other dementias or degenerative diseases. It would be worthwhile to follow up the current work with samples from other types of dementia to address the question of individual protein's specificity to the pathology of interest.

Although confident identification of around 10% of human proteome indicates the analytical sensitivity of the current experimental design, cellular or subcellular localization and distribution of the regulated proteins among different cell-types are not clear from our experiment. We tried to partially address this by selecting relatively cell-type specific candidates (e.g. PEA15, SYNPO, syntaxin) for data interpretation and validation. Immunohistochemical studies on similar samples targeting some of the regulated candidates will be complementary to this approach.

## 6 Conclusions

Our quantitative clinical proteomic study demonstrates deregulated proteome of the temporal cortex of VaD patients compared to non-demented control subjects. We have successfully applied novel sample preparation methods compatible with the systems approach to confidently identify 2281 proteins by iTRAQ experiment using a group-wise sample pooling strategy. The majority of the deregulated proteome has never been reported in context of VaD or dementia. In addition, we were able to obtain statistically significant difference of three candidate proteins (SOD1, NCAM and ATP5A) between VaD and control brain based on the WB analysis of twenty individual subjects. Some of the key proteins that play crucial roles in the progressive parenchymal and vascular changes during VaD, like cortical hypoperfusion, hypometabolism, inflammation and oxidative stress were identified in a single experiment. The parallel aberrant regulation of multiple proteins may indicate an early degenerative changes or an actively ongoing adaptive process that can only be captured by a systems biological tool like proteomics. The tissue samples represent the most proximal source to determine the pathologic alteration of proteome that could eventually be reflected in the CSF or plasma. Thus, the up-regulated proteome could provide a list of potential candidates for future exploration of biomarkers from body fluids by targeted analysis in clinically similar cohorts of VaD patients. Detailed understanding of these pathologic events and their correlation with dementia and cerebrovascular pathology through follow-up studies could provide potential therapeutic targets for VaD.

## Conflict of interest

None for this study.

## Figures and Tables

**Table 1:** Demographics and disease variables for groups of control and VaD patients.

Demographics		Control	VaD
	Number of cases	10	10
	Age at death (mean yrs ± SD)^a^	80.3 ± 8.9	84.0 ± 8.5
	Sex (male/female)	2 M/8 F	7 M/3 F
	Duration of illness (yrs)	na	3.8 ± 3.5
	Post-mortem interval (median ± IQR)^a^	24.5 ± 24.0	36.5 ± 41.0
Disease variables			
	Pre-death MMSE (median ± SD/N)	na	14.3 ± 3.7(6)
Braak staging (N)	0–II	5	9
	III–IV	3	0
	V–VI	0	0
	NA*	2	1

SD, standard deviation; IQR, interquartile range. na, not applicable; *NA, Not available.

a The groups were not significantly different in terms of Age (independent sample *t*-test, *p*-value > 0.05) or post-mortem interval (Mann–Whitney *U* test, *p*-value > 0.05).

**Table 2:** Significantly regulated proteome of VaD selected from the complete list of confidently identified proteins^a^.

N	Unused	%Cov (95)	Gene symbol	Name	Peptides (95%)	VaD: Control (115:114)	EF^†^ (115:114)
**Energy metabolism**							
Glycolysis							
16	214.6	96.1	ENO1	Alpha-enolase	517	1.74	1.15
18	212.7	99.7	ALDOA	Fructose-bisphosphate aldolase A	421	0.53	1.19
27	179.8	99.8	PGK1	Phosphoglycerate kinase 1	296	0.47	1.31
43	124.9	100.0	ALDOC	Fructose-bisphosphate aldolase C	298	0.54	1.20
150	56.2	66.8	LDHB	l-Lactate dehydrogenase B chain	74	0.60	1.16
311	33.7	64.2	LDHA	l-Lactate dehydrogenase A chain	30	0.52	1.29
Pyruvate dehydrogenase complex							
332	32.2	31.3	PDHA1	Pyruvate dehydrogenase E1 component subunit alpha, somatic form, mitochondrial	19	0.61	1.19
355	30.5	49.9	PDHB	Pyruvate dehydrogenase E1 component subunit beta, mitochondrial	37	0.58	1.21
395	27.2	29.5	DLAT	Dihydrolipoyllysine-residue acetyltransferase component of pyruvate dehydrogenase complex, mitochondrial	20	0.34	1.57
Tricarboxylic acid cycle							
51	113.1	68.1	ACO2	Aconitate hydratase, mitochondrial	139	0.46	1.38
118	65.2	60.6	IDH2	Isocitrate dehydrogenase [NADP], mitochondrial	39	0.35	1.45
222	42.8	63.9	FH	Fumarate hydratase, mitochondrial	40	0.44	1.50
253	39.1	56.6	IDH3A	Isocitrate dehydrogenase [NAD] subunit alpha, mitochondrial	26	0.62	1.19
324	32.7	40.2	SUCLG1	Succinyl-CoA ligase [ADP/GDP-forming] subunit alpha, mitochondrial	35	0.49	1.31
565	20.0	31.5	SUCLA2	Succinyl-CoA ligase [ADP-forming] subunit beta, mitochondrial	12	0.51	1.26
Oxidative phosphorylation							
41	130.1	72.7	**ATP5A1**	ATP synthase subunit alpha, mitochondrial	124	0.57	1.26
42	129.9	80.5	ATP5B	ATP synthase subunit beta, mitochondrial	191	0.52	1.20
168	51.9	77.8	ATP5J	ATP synthase-coupling factor 6, mitochondrial	64	1.75	1.19
178	49.9	40.4	NDUFS1	NADH-ubiquinone oxidoreductase 75 kDa subunit, mitochondrial	35	0.49	1.36
193	47.6	55.4	DLD	Dihydrolipoyl dehydrogenase, mitochondrial	29	0.53	1.26
202	45.6	61.6	**UQCRC2**	Cytochrome b–c1 complex subunit 2, mitochondrial	36	0.53	1.36
274	37.7	78.9	ATP5O	ATP synthase subunit O, mitochondrial	29	0.51	1.45
340	31.8	45.7	**SDHB**	Succinate dehydrogenase [ubiquinone] iron–sulfur subunit, mitochondrial^b^	22	0.72	1.13
1232	6.2	19.4	**MT-CO2**	Cytochrome c oxidase subunit 2^b^	4	1.06	1.21
1260	6.1	28.0	**NDUFB8**	NADH dehydrogenase [ubiquinone] 1 beta subcomplex subunit 8, mitochondrial^b^	4	0.95	1.15
**Vascular dysfunction and inflammation**							
17	214.3	92.1	ALB	Serum albumin	235	2.03	1.29
30	161.4	100.0	HBB	Hemoglobin subunit beta	356	0.26	1.39
126	62.3	31.3	L1CAM	Neural cell adhesion molecule L1	56	1.49	1.12
157	53.7	38.0	**NCAM1**	Neural cell adhesion molecule 1	37	1.79	1.32
256	38.7	31.8	**ICAM5**	Intercellular adhesion molecule 5	31	1.75	1.21
265	38.1	27.8	FGA	Fibrinogen alpha chain	25	2.23	1.53
326	32.5	57.8	IGHA1	Ig alpha-1 chain C region	24	5.55	2.36
383	28.3	47.5	IGHG2	Ig gamma-2 chain C region	20	4.02	1.84
419	26.0	96.6	HBD	Hemoglobin subunit delta	152	0.54	1.21
442	25.2	26.8	IGSF8	Immunoglobulin superfamily member 8	15	2.13	1.46
461	24.2	47.9	FGB	Fibrinogen beta chain	15	1.94	1.46
609	18.4	74.5	IGLC2	Ig lambda-2 chain C regions	13	3.77	1.96
734	14.5	90.6	IGKC	Ig kappa chain C region	20	4.41	2.17
1256	6.1	3.0	COL6A3	Collagen alpha-3(VI) chain	8	1.79	1.29
**Synaptic transmission**							
9	266.6	99.8	ENO2	Gamma-enolase	586	1.58	1.14
26	181.9	98.4	PEBP1	Phosphatidylethanolamine-binding protein 1	342	1.57	1.17
119	65.0	39.9	ATP1A3	Sodium/potassium-transporting ATPase subunit alpha-3	45	2.96	1.53
121	63.7	80.1	RPS27A	Ubiquitin-40S ribosomal protein S27a	55	3.87	1.74
133	60.1	43.8	**SYNPO**	Synaptopodin^b^	46	0.73	1.10
141	58.2	52.8	PPP3CA	Serine/threonine-protein phosphatase 2B catalytic subunit alpha isoform	52	0.57	1.27
218	43.3	49.2	ABAT	4-Aminobutyrate aminotransferase, mitochondrial	26	0.59	1.21
244	40.1	40.5	ALDH9A1	4-Trimethylaminobutyraldehyde dehydrogenase	29	0.56	1.26
345	31.6	65.0	SNAP25	Synaptosomal-associated protein 25	34	1.64	1.20
358	30.3	36.5	**STX1B**	Syntaxin-1B	23	1.89	1.29
463	24.1	14.3	SLC1A2	Excitatory amino acid transporter 2	22	2.63	1.51
667	16.5	29.5	**STX1A**	Syntaxin-1A	9	1.85	1.26
783	13.3	26.0	ATP1A2	Sodium/potassium-transporting ATPase subunit alpha-2	32	2.21	1.60
**Oxidative stress**							
63	99.0	99.3	**SOD1**	Superoxide dismutase [Cu–Zn]	188	3.02	1.72
134	59.8	82.5	**FTH1**	Ferritin heavy chain	135	2.05	1.41
152	55.4	63.4	**FTL**	Ferritin light chain	80	2.21	1.41
208	44.5	85.7	PRDX6	Peroxiredoxin-6	48	0.41	1.45
279	37.2	84.7	GPX1	Glutathione peroxidase 1^b^	30	1.18	1.13
898	11.0	14.2	GSR	Glutathione reductase, mitochondrial^b^	8	0.63	1.26
**Chaperonic function**							
70	90.8	60.9	HSPA5	78 kDa glucose-regulated protein	94	1.42	1.12
72	88.6	61.1	HSP90AA1	Heat shock protein HSP 90-alpha	79	0.43	1.45
92	76.2	67.2	STIP1	Stress-induced-phosphoprotein 1	61	1.45	1.15
132	60.2	53.5	**HSPA4**	Heat shock 70 kDa protein 4	40	0.46	1.42
255	38.7	96.1	HSPB1	Heat shock protein beta-1	63	1.77	1.17
330	32.3	44.0	CCT8	T-complex protein 1 subunit theta	19	1.80	1.29
656	17.0	80.6	TBCA	Tubulin-specific chaperone A	16	1.57	1.20
**Others**							
71	90.6	37.6	NFASC	Neurofascin	72	1.75	1.26
73	87.9	96.1	**VDAC1**	Voltage-dependent anion-selective channel protein 1	91	0.56	1.17
84	80.6	100	SNCG	Gamma-synuclein^b^	91	1.08	1.04
200	46.0	33.4	NPEPPS	Puromycin-sensitive aminopeptidase	27	0.42	1.47
203	45.4	97.7	**PEA15**	Astrocytic phosphoprotein PEA-15	42	1.56	1.15
651	17.2	15.1	PREP	Prolyl endopeptidase	10	0.58	1.20
799	12.8	37.4	PSMA6	Proteasome subunit alpha type-6	8	0.64	1.26

The gene symbols of the proteins selected for WB validation are shown in bold. The proteins have been classified based on their respective primary function/pathway. The energy metabolism category is sub-divided into four groups. Majority of the proteins related to energy metabolism were down-regulated, while proteins related to inflammation (immune response and cell adhesion) were up-regulated. Proteins participating in synaptic transmission and chaperonic function displayed a mixed trend.

a The list of proteins qualified through the preset selection criteria (unused prot score > 2.0, p < 0.05, regulation cut-off : 1.4 fold compared to the control group) along with their relative quantitative values. The gene symbols of the proteins selected for WB validation are shown in bold. The proteins have been classified based on their respective primary function/pathway. The energy metabolism category is sub-divided into four groups. Majority of the proteins related to energy metabolism were down-regulated, while proteins related to inflammation (immune response and cell adhesion) were up-regulated. Proteins participating in synaptic transmission and chaperonic function displayed a mixed trend.

b Proteins incorporated in the list due to their close association with the regulated proteins although they did not meet the above-mentioned preset selection criteria. For example, the fold of down-regulation was < 1.4 fold for SYNPO, while others (e.g. SDHB, SNCG) doesn't have a significant *p*-value (i.e. *p* > 0.05).

## Appendix A Supplementary data

**Supplemental Table 1**. Demographics and Disease Variables of Individual Patients for Groups of Control and VaD patients. **Supplemental Table 2**. Complete information of the full list of the qualified proteins obtained from the bias and background corrected iTRAQ data set. **Supplemental Table 3**. The List of Up- and Down-regulated Proteins Short-listed Following a Two-step Filtering (*p*-value < 0.05, cut-off = 1.4 fold) of the Complete List of Confidently Identified Proteins. **Supplemental Table 4**. Significant Correlations obtained through Pearson's product moment Correlation Performed Individually on VaD and Control Groups as well as on Combined VaD/Control Group.
